# A mechanosensitive gene signature associated with immune landscape and survival in glioma: a multi-cohort analysis

**DOI:** 10.1186/s42466-026-00515-2

**Published:** 2026-08-07

**Authors:** Tianxue Yang, Ye Yuan, Nianhong Lu, Rui Han, Ziran Xu

**Affiliations:** 1 Department of Clinical Laboratory, Lequn Branch, The First Hospital of Jilin University, Changchun, Jilin China; 2 Department of Colorectal and Anal Surgery, General Surgery Center, The First Hospital of Jilin University, Changchun, Jilin China; 3 Department of Medical Oncology, West German Cancer Center, University Hospital Essen, 45147 Essen, Germany; 4 Department of Neurovascular Surgery, The First Hospital of Jilin University, Changchun, Jilin China

**Keywords:** Glioma, Mechanosensitive genes, Immune landscape, Prognostic signature, Tumor microenvironment

## Abstract

**Background:**

Glioma is characterized by substantial molecular heterogeneity and poor clinical outcomes. Emerging evidence suggests that mechanotransduction-related pathways may influence tumor progression and immune remodeling. However, the prognostic relevance of mechanosensitive genes in glioma remains unclear.

**Methods:**

RNA-sequencing data and clinical information from the TCGA, CGGA-301, CGGA-325, and GSE16011 cohorts were analyzed. Mechanosensitive genes were identified from curated gene sets, and a prognostic signature was constructed using LASSO and Cox regression analyses. Immune infiltration, immune checkpoint expression, TIDE predictions, and drug sensitivity were evaluated. The risk score was consistently and positively correlated with SPP1 expression across four independent cohorts, and single‑cell analysis further suggested that SPP1‑related signaling may contribute to immune microenvironment remodeling. Selected genes were validated by qRT-PCR in glioma cell lines.

**Results:**

A nine-gene mechanosensitive signature stratified glioma patients into high- and low-risk groups with significantly different overall survival across multiple cohorts. The risk score was independently associated with established clinicopathological factors, including WHO grade, IDH mutation status, 1p/19q co-deletion status, and MGMT promoter methylation status. High-risk tumors exhibited increased macrophage and neutrophil infiltration, elevated immune checkpoint expression, and higher predicted immune evasion potential. Single-cell analysis suggested that SPP1-related signaling may contribute to immune microenvironment remodeling. Experimental validation supported differential expression of selected genes in glioma cells.

**Conclusions:**

This mechanosensitive gene signature is associated with immune landscape heterogeneity and survival in glioma. The model may provide complementary stratification information and support further investigation of mechanobiology-related tumor–microenvironment interactions.

**Graphical abstract:**

**Figure:**
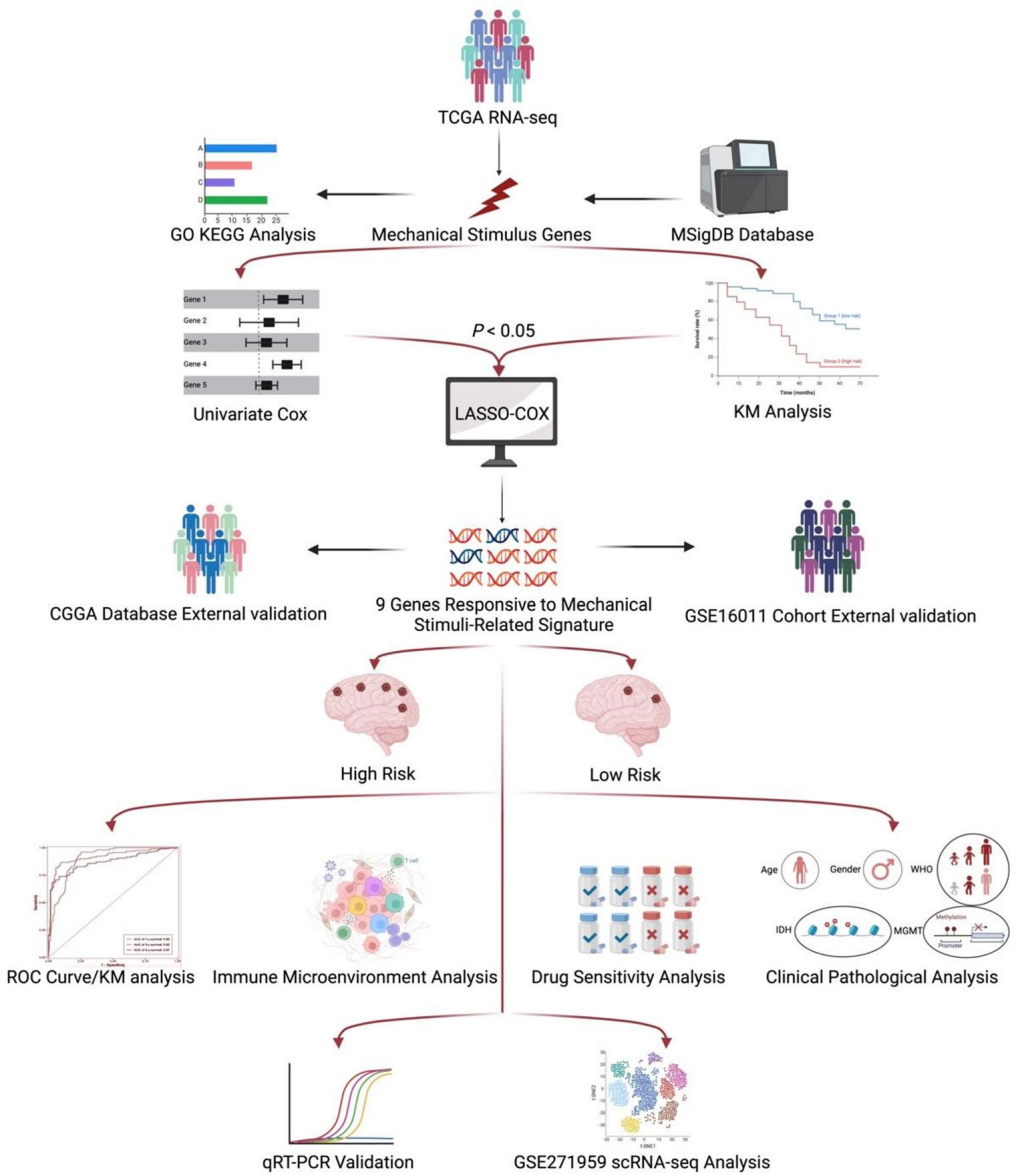

**Supplementary Information:**

The online version contains supplementary material available at 10.1186/s42466-026-00515-2.

## Introduction

Glioma is the most common and deadly primary malignant brain tumor of the central nervous system, accounting for approximately 80% of malignant brain tumors. Despite significant advances in traditional glioma treatments, such as surgery, radiotherapy, and chemotherapy, the highly invasive and malignant nature of gliomas, combined with limited treatment options [1], means that the overall prognosis for most glioma patients remains poor [2]. The average survival time is only 12–15 months [3, 4], with nearly 80% of patients dying within the first year after diagnosis [5]. Beyond tumor progression, neurological complications such as status epilepticus have been associated with functional decline and high mortality in patients with brain tumors, highlighting the broader clinical burden of tumor-related neurological events [6]. Therefore, it is crucial to improve molecular risk stratification and understand tumor–microenvironment interactions.

One of the main reasons for glioma recurrence and poor prognosis is the infiltration of glioma cells into healthy brain tissue [7]. Glioma cells can spread extensively, even to the contralateral hemisphere, making these invasive glioma cells difficult to eradicate surgically [8]. The invasion of glioma is influenced by numerous complex factors within the tumor microenvironment (TME), including mechanical stress [9]. In addition to affecting invasion, the mechanical heterogeneity of microregions in the brain may alter cellular transcriptional programs, pushing cells toward a more aggressive mesenchymal phenotype [10]. The biomechanical properties of the TME can also regulate glioma proliferation, survival, migration, and drug response by influencing metabolism [11, 12]. This suggests that the mechanical properties of gliomas play a fundamental role in disease progression, immune evasion, and treatment response [13]. However, the underlying molecular mechanisms are just beginning to be discovered [12]. Therefore, identifying new therapeutic targets from the perspective of mechanical forces may be key to discovering new treatment strategies for glioma.

Brain tissue is soft and sensitive, providing a microenvironment with low mechanical resistance. Cancer cells may adapt to the specific mechanical properties of the brain through both intrinsic genetic mechanisms and extrinsic microenvironmental cues [14]. Previous studies have reported that the mechanical modulus of the glioma TME increases with disease progression [15–17]. While it is generally believed that gliomas are stiffer than brain tissue, the stiffness varies significantly across different regions of the glioma, suggesting that different regions of gliomas may have distinct mechanical properties. Moreover, solid stress, interstitial fluid pressure, and extracellular matrix (ECM) stiffness influence tumor behavior, making it challenging to explore the impact of mechanical properties on gliomas in vitro [13]. Currently, there is a lack of preclinical research in this area, and using mechanical gene set models for analysis may be an important way to avoid complex confounding factors. Precision oncology holds great potential in addressing glioma detection issues [18, 19], but few studies have systematically analyzed the impact of mechanical forces on tumors. This approach could help identify key mechanical signals that affect poor prognosis and immune characteristics in gliomas, making it an ideal pathway to improving survival rates.

In this study, we established a mechanosensitive nine-gene risk signature and systematically evaluated its associations with clinical outcome, immune landscape, therapeutic response, and tumor–microenvironment interactions using multiple independent cohorts and single-cell transcriptomic analyses.

## Methods

### Data collection

We downloaded comprehensive RNA-seq data for lower-grade glioma (LGG) and glioblastoma (GBM) from The Cancer Genome Atlas (TCGA) the UCSC Xena website (https://xena.ucsc.edu/). After excluding samples lacking clinical information, data from 691 patients with glioma were included. The GSE16011 cohort was derived from the Gene Expression Omnibus (GEO) database (http://www.ncbi.nlm.nih.gov/geo/). The clinical information was obtained from a published study on the intrinsic gene expression profiles of gliomas [20]. Additionally, we collected mRNA expression data and corresponding clinicopathological characteristics from the CGGA-301 and CGGA-325 cohorts within the Chinese Glioma Genome Atlas (CGGA) (http://www.cgga.org.cn). Genes related to the response to mechanical stimuli were identified using the Molecular Signatures Database (https://www.gsea-msigdb.org/gsea/login.jsp).

### Functional enrichment analysis

To further explore the functions of the genes related to the response to mechanical stimuli, we conducted Gene Ontology (GO) enrichment analysis to examine their enrichment in biological process categories. Additionally, Kyoto Encyclopedia of Genes and Genomes (KEGG) enrichment analysis was performed to investigate the pathways enriched by these genes. Both analyses were conducted using the clusterProfiler package, with visualizations created using the enrichplot package. A p-value < 0.05 was considered statistically significant.

### Univariate cox regression and survival analysis

We performed univariate Cox regression and Kaplan–Meier (KM) survival analyses for genes related to the response to mechanical stimuli in the TCGA cohort using the survival R package. Genes significantly associated with overall survival (*p* < 0.05) were included in subsequent analyses.

### Model construction and validation

The genes related to the response to mechanical stimuli with prognostic value were further analyzed using least absolute shrinkage and selection operator (LASSO) regression and stepwise Cox regression in the TCGA cohort to establish a risk score model. A forest plot was generated using the “survminer” package in R. The risk score for each patient was calculated as follows: *Risk score*=$\:{\sum\:}_{i=1}^{N}({Coef}_{i},\:\times\:,{Exp}_{i})$, where ‘i’ denoted the gene, EXPi represented the expression level of the genes related to the response to mechanical stimuli, and Coefi was the corresponding regression coefficient derived from multivariate Cox regression analysis. The median risk score was used to stratify patients into high-risk and low-risk groups. The TCGA cohort served as the training set, with external validation performed using the CGGA cohort and the GSE16011 dataset.

### Survival analysis

To assess the OS of glioma patients across different groups, we conducted KM survival analyses using the survival and survminer R packages. The accuracy of the risk score in predicting survival outcomes was evaluated using time-dependent receiver operating characteristic (ROC) curves generated with the survival ROC package in R. A larger area under the ROC curve indicated stronger predictive ability. The pheatmap package was utilized to visualize the survival status of samples in different risk groups.

### Independent prognostic value of risk signature and nomogram construction

To exclude the influence of other clinicopathological factors, such as tumor grade, gender, age, isocitrate dehydrogenase (IDH) mutation status, 1p/19q codeletion status, and MGMT promoter methylation status, on the predictive ability of the mechanosensitive risk signature, we conducted univariate and multivariate Cox regression analyses using the survival R package. The results were presented as a forest plot generated with the forestplot R package.

To better understand the relative contributions of variables in the multivariate Cox regression analysis, we used the survival and rms packages in R to construct a nomogram combining patients’ clinical characteristics with mechanosensitive risk scores. To evaluate the predictive performance of the nomogram and compare it with decision curves from other independent prognostic factors, we plotted calibration curves and performed decision curve analysis (DCA).

### Immune microenvironment analysis and immunotherapy response

To investigate immune cell infiltration in tumor samples from high-risk and low-risk groups, we employed the CIBERSORT algorithm. The infiltration levels of 22 immune cell types between the two risk groups were visualized using box plots. We also analyzed mRNA expression levels of immune checkpoint molecules and immunosuppressive factors in the TCGA cohort, comparing high-risk and low-risk groups. To further assess the potential response to immune checkpoint inhibitors (ICIs) therapy, we predicted responses in different risk groups using the Tumor Immune Dysfunction and Exclusion (TIDE) online tool (http://tide.dfci.harvard.edu/), with TIDE scores, immune dysfunction, immune exclusion, and microsatellite instability (MSI) scores displayed as box plots.

### Drug sensitivity analysis

Drug sensitivity analysis was conducted by using established gene expression profiles from cell lines and corresponding drug sensitivity data to predict the sensitivity of other samples to specific drugs. The oncoPredict R package provided two commonly used drug-response training datasets: the Cancer Therapeutics Response Portal V2 and the Genomics of Drug Sensitivity in Cancer V2 (GDSC-V2). We used GDSC-V2 as the training set to estimate the predicted IC50 values for patients in the TCGA cohort. The IC50 value, indicative of drug sensitivity, represented the drug concentration required to inhibit cell viability by 50%, with lower IC50 values indicating higher sensitivity. The IC50 values of eight commonly used anticancer drugs for glioma patients (temozolomide, vincristine, carmustine, cisplatin, ribociclib, irinotecan, erlotinib, palbociclib) were evaluated using the Wilcoxon test, and differences between groups were visualized using box plots generated by the “ggpubr” and “ggplot2” R packages. To explore correlations between mechanosensitive genes and drugs, we created a correlation heatmap using the pheatmap R package.

### Single-cell data processing and intercellular communication

Single-cell RNA-sequencing data from three glioma tissue samples in the GSE271959 dataset were obtained from the GEO database (https://www.ncbi.nlm.nih.gov/geo/). Seurat objects were created using the Seurat R package, with cells exhibiting abnormal mitochondrial gene proportions excluded, resulting in 37,090 cells being included for further analysis. Batch effects among samples were corrected using the Harmony R package. Dimensionality reduction was performed using the uniform manifold approximation and projection algorithm. Cell types were identified through manual annotation, with marker genes primarily sourced from the CellMarker database (http://xteam.xbio.top/CellMarker). Subsequently, an intercellular communication network was conducted using the CellChat R package.

### Cell lines and culture conditions

The normal human astrocyte cell line NHA and the glioma cell lines U87 and U251 were obtained from the American Type Culture Collection (ATCC). The cells were maintained in DMEM (BBI, E600003) supplemented with 10% foetal bovine serum (BBI, E600001) and 1% penicillin-streptomycin (Gibco, 15140122) at 37 °C with 5% CO₂.

### RNA isolation and quantitative reverse transcription PCR (qRT-PCR)

Total RNA was isolated using TransZol Up (TransGen Biotech, ET111-01-V2) according to the manufacturer’s instructions, and according to the process for Hifair™ III 1st Strand cDNA Synthesis SuperMix for qPCR (gDNA Digester Plus) (Yeasen, 11141ES60) to obtain cDNA. We then used specific primers and Hieff^®^ qPCR SYBR^®^ Green Master Mix (High Rox) (Yeasen, 11203ES03) in the Applied Biosystems 7300 Real-Time PCR System for qRT-PCR. Relative expression levels were expressed relative to those in Normal Human Astrocytes (NHA). The housekeeping gene *GAPDH* was used as an internal reference control. The primer sequences used are listed in Supplementary Table 1. Quantitative data are presented as mean ± SEM of three independent experiments.

### Statistical analysis

All statistical analyses were conducted using R (version 4.2.1) and GraphPad Prism 10. LASSO and Cox regression analyses were used to identify prognostic genes. KM analysis was performed to evaluate survival outcomes, and Spearman’s correlation analysis was conducted to assess correlations. The Wilcoxon test was used for comparisons between two groups, while one-way ANOVA and Dunnett’s multiple comparisons test, as well as the Kruskal-Wallis H test, were used for comparisons among multiple groups. A *p-value * < 0.05 was considered statistically significant, and graphical representations were created using R software or GraphPad Prism.

## Results

### Identification of prognostic genes responsive to mechanical stimuli in the TCGA cohort and functional enrichment analysis

**Fig. 1:**
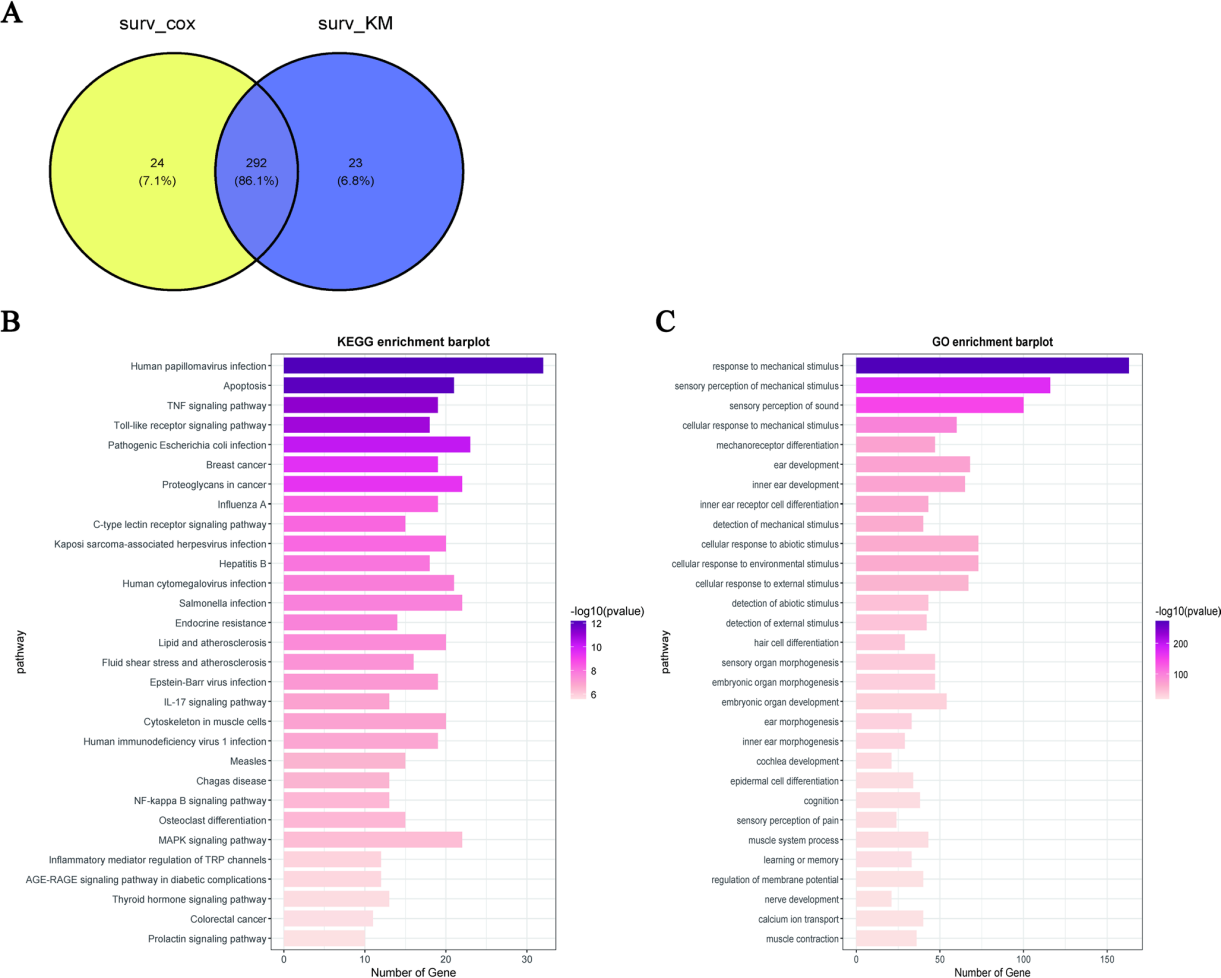
Biological functions of mechanically related prognostic genes in the Cancer Genome Atlas (TCGA) cohort. (**A**) Significant intersecting genes from the mechanical stimuli-related gene set identified through univariate Cox regression and Kaplan-Meier analysis in the TCGA database. (**B**) Top 30 Kyoto Encyclopedia enriched pathways and (**C**) biological process entries of Gene Ontology analysis enriched in the intersecting genes

The methodological workflow included a total of 691 glioma samples from the TCGA cohort, 301 glioma samples from the CGGA 301 cohort, 324 glioma samples from the CGGA 325 cohort, and 276 glioma samples from the GSE16011 cohort, all of which had complete clinicopathological information. Detailed information about these samples was provided in Supplementary Tables 2, 3, 4, and 5. We extracted a total of 399 mechanosensitive genes from five gene sets listed in Supplementary Table 6 to generate prognostic gene signatures. These genes were subjected to univariate Cox regression and KM analyses, resulting in the identification of 292 survival-related mechanosensitive prognostic genes (*p* < 0.05, Fig. 1A). GO enrichment analysis of these genes indicated that the primary enriched terms were biological processes related to the response to mechanical stimuli, including response to mechanical stimulus, sensory perception of mechanical stimulus, sensory perception of sound, cellular response to mechanical stimulus, etc., as well as processes involved in the development of mechanoreceptive cells or organs, such as mechanoreceptor differentiation, ear development, sensory organ morphogenesis, nerve development, etc. (Fig. 1B). These findings further illustrated that these genes were key mediators in the human body’s response to external mechanical stimuli. KEGG pathway analysis suggested that these genes were associated with pathways related to viral and bacterial infections and immune-related signalling pathways, such as human papillomavirus infection, TNF signalling pathway, Toll-like receptor signalling pathway, pathogenic *Escherichia* *coli* infection, etc. (Fig. 1C).

### Construction of a novel prognostic signature based on genes responsive to mechanical stimuli in the TCGA cohort

**Fig. 2:**
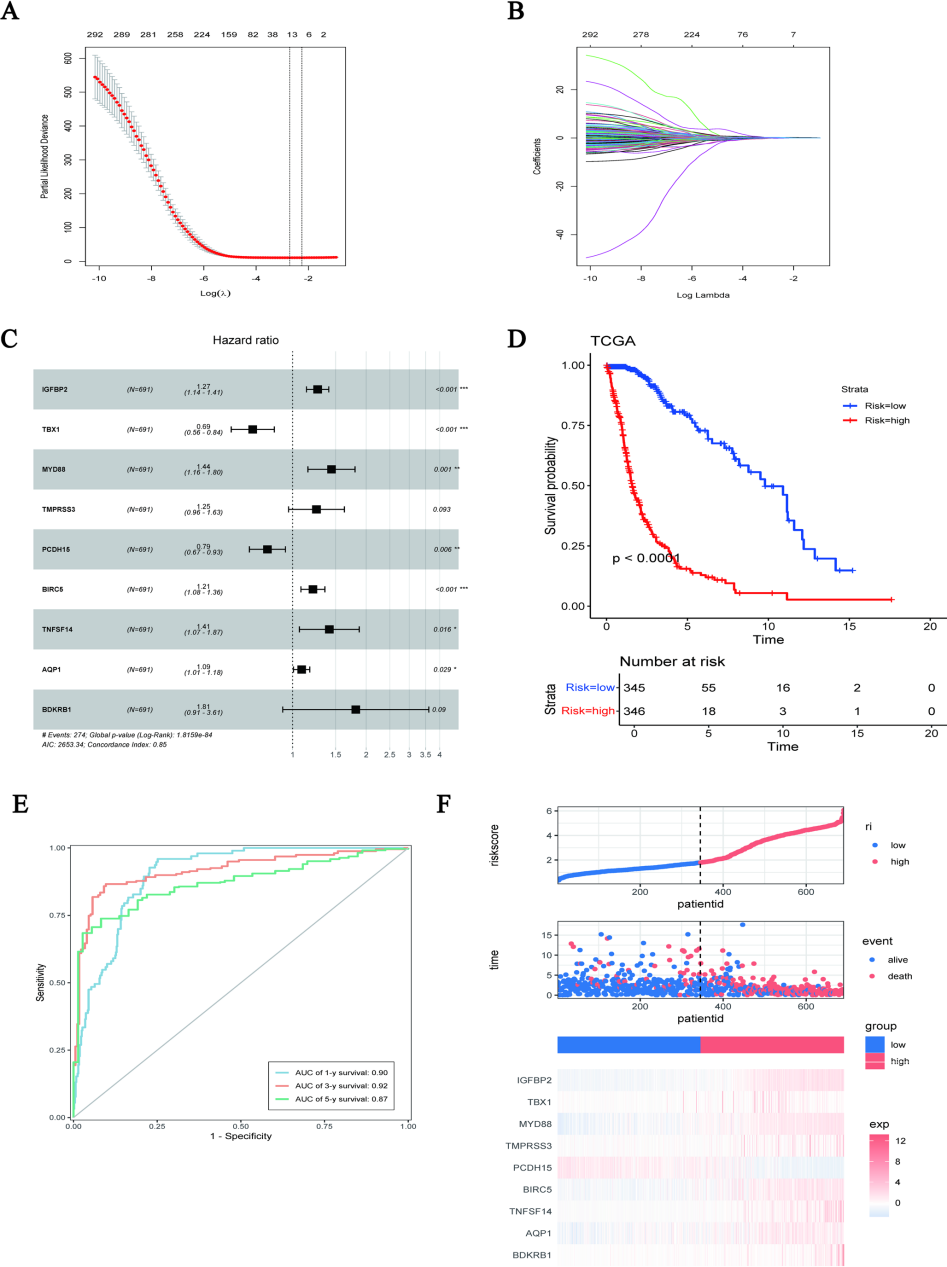
Evaluation of prognostic predictive power of genes related to the response to mechanical stimuli based on the Cancer Genome Atlas (TCGA) cohort data. (**A**) Least absolute shrinkage and selection operator (LASSO) coefficient distribution of 292 genes related to the response to mechanical stimuli in the TCGA dataset. (**B**) Selection of the optimal parameter (lambda) in the LASSO model. (**C**) Nine genes were selected to establish the prognostic signature. (**D**) Kaplan-Meier curves for different groups in the TCGA glioma cohort. (**E**) Time-dependent receiver operating characteristic curves and area under the curves validating the predictive efficacy of the risk score. (**F**) Distribution of risk scores and median values in the TCGA cohort; distribution of survival time, survival status, and risk scores; distribution of risk scores, clinical characteristics, and gene expression

The 292 genes identified (Supplementary Table 7) were subjected to LASSO regression analysis to avoid overfitting issues in the risk signature (Fig. 2A, B). A multivariate Cox regression analysis using the AIC method was then applied to the genes returned by the LASSO regression (16 genes), resulting in the construction of an optimal model comprising 9 genes (*IGFBP2*, *TBX1*, *MYD88*, *TMPRSS3*, *PCDH15*, *BIRC5*, *TNFSF14*, *AQP1*, and *BDKRB1*) (Fig. 2C). Among these, *TBX1* and *PCDH15* were identified as protective factors for glioma survival, with hazard ratios (HRs) < 1, while *IGFBP2*, *MYD88*, *TMPRSS3*, *BIRC5*, *TNFSF14*, *AQP1*, and *BDKRB1* were identified as risk factors for glioma survival, with HRs > 1. Based on the expression levels of these 9 genes and their corresponding regression coefficients, the following risk score formula was constructed: Risk score = 0.23615$\:\times\:$*IGFBP2**\:-*0.37548$\:\times\:$*TBX1**\:+*0.36628$\:\times\:$*MYD88**\:+*0.22497$\:\times\:$*TMPRSS3**\:-*0.23758$\:\times\:$*PCDH15**\:+*0.19160$\:\times\:$*BIRC5**\:+*0.34602$\:\times\:$*TNFSF14**\:+*0.08519$\:\times\:$*AQP1**\:+*0.59570$\:\times\:$*BDKRB1*. To assess the prognostic prediction performance of this gene signature, the risk score for each sample was calculated using the formula mentioned above. Subsequently, glioma patients in the TCGA cohort were divided into high-risk (*n* = 345) and low-risk (*n* = 346) groups based on the median risk score. Compared to low-risk patients, high-risk patients exhibited earlier disease progression and poorer survival outcomes. Additionally, KM survival analysis showed that the OS rate was significantly lower in the high-risk group than in the low-risk group (*p* < 0.0001, Fig. 2D). To further evaluate the predictive accuracy of the risk score, time-dependent ROC curves were constructed. The area under the curve (AUC) for OS at 1, 3, and 5 years reached 0.90, 0.92, and 0.87, respectively (Fig. 2E). Furthermore, Fig. 2F showed the distribution of the OS-related predictive model in the TCGA dataset.

### Validation of the prognostic signature of genes responsive to mechanical stimuli in the CGGA and GSE16011 cohorts

**Fig. 3:**
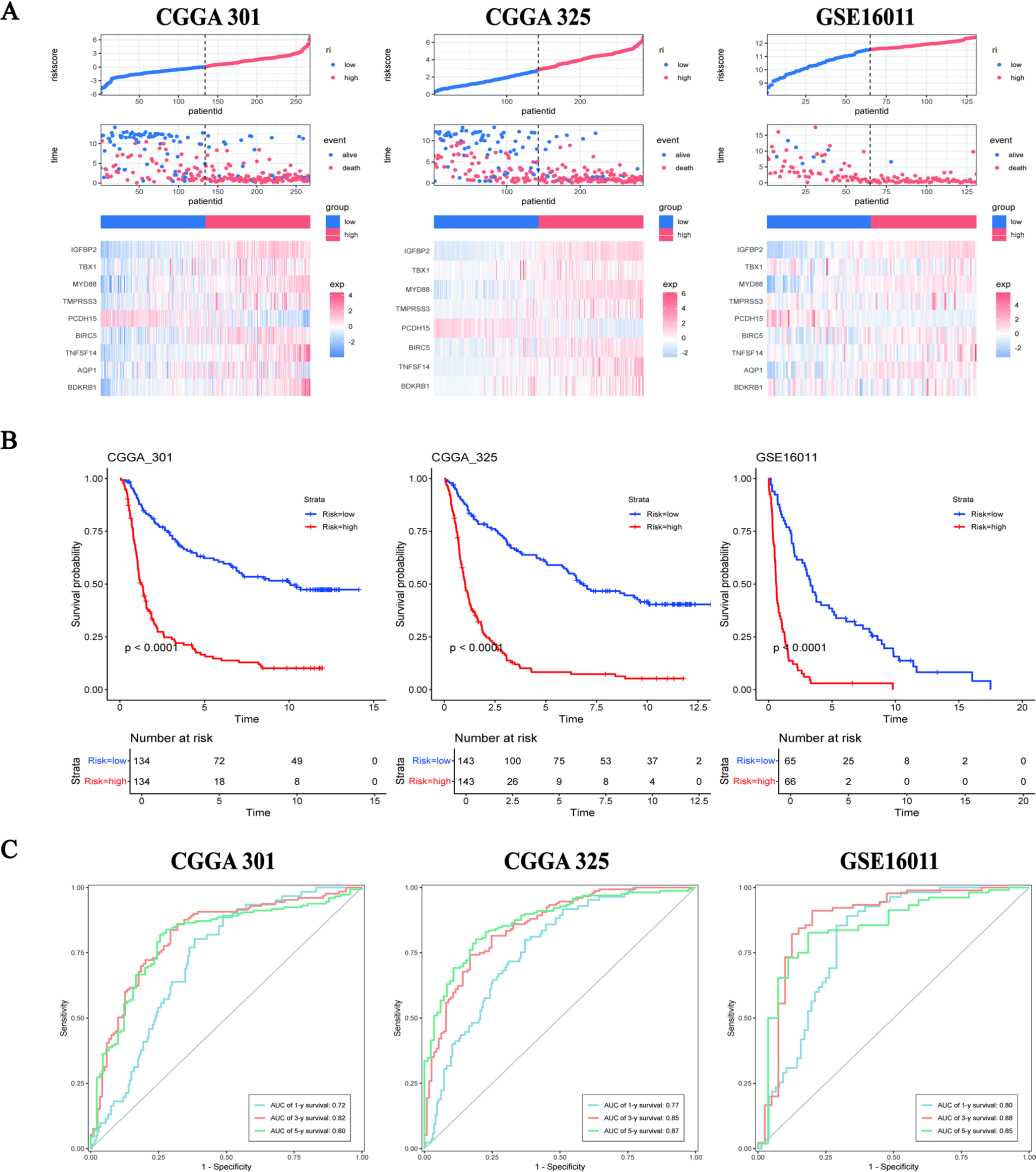
External validation of the prognostic performance of mechanical stimuli-related gene signature in the Chinese Glioma Genome Atlas (CGGA) 301, CGGA 325, and GSE16011 Cohorts. (**A**) Distribution of risk scores and median values in the CGGA 301, CGGA 325, and GSE16011 cohorts; distribution of survival status, survival time, and risk scores; distribution of risk scores, clinical characteristics, and gene expression. (**B**) Kaplan-Meier curves for overall survival in different groups based on the cutoff points determined from the Cancer Genome Atlas cohort in the CGGA 301, CGGA 325, and GSE16011 cohorts. (**C**) Time-dependent receiver operating characteristic curves and area under the curves for the CGGA 301, CGGA 325, and GSE16011 cohorts

To validate the robustness and reproducibility of the mechanistically related prognostic features developed using the TCGA cohort for predicting OS in glioma, we first applied the scoring formula from the TCGA cohort to calculate risk scores for glioma patients in the CGGA 301, CGGA 325, and GSE16011 cohorts. Patients in each cohort were then divided into high-risk and low-risk groups based on the median risk score. As expected, similar results were observed across all three cohorts, with patients in the high-risk group exhibiting a significantly higher risk of death compared to those in the low-risk group (*p* < 0.0001, Fig. 3A, B). Moreover, in the CGGA 301 cohort, the AUC for OS was 0.72, 0.81, and 0.80 at 1, 3, and 5 years, respectively; in the CGGA 325 cohort, 0.90, 0.92, and 0.87; and in the GSE16011 cohort, 0.80, 0.88, and 0.85 (Fig. 3C). These findings confirmed the reproducibility of the risk model in three independent external cohorts.

### The mechanosensitive signature is associated with clinicopathological features

**Fig. 4:**
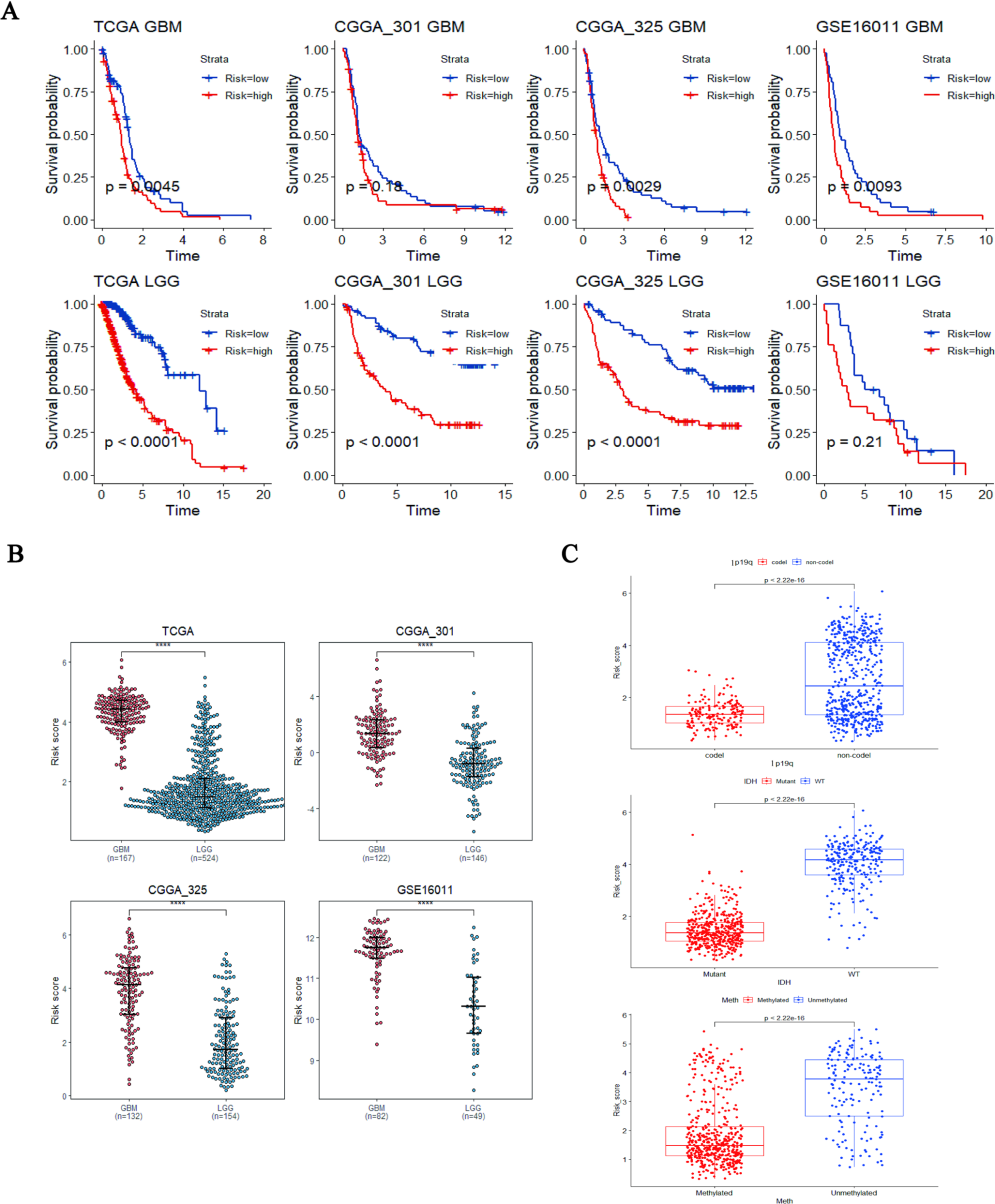
Mechanical feature risk scores across different glioma clinicopathological characteristics. (**A**) Prognostic value of the mechanically related risk model for glioblastoma (GBM) and low-grade glioma (LGG) in the Cancer Genome Atlas (TCGA), Chinese Glioma Genome Atlas (CGGA) 301, CGGA 325, and GSE16011 datasets. (**B**) Distribution of risk scores for LGG and GBM in the TCGA, CGGA 301, CGGA 325, and GSE16011 datasets (Wilcoxon test). (**C**) Distribution of risk scores in the TCGA dataset stratified by 1p/19q co-deletion status, isocitrate dehydrogenase mutation status, and MGMT promoter methylation status (Wilcoxon test)

We further explored whether the mechanosensitive risk signature is associated with the clinicopathological characteristics of glioma patients. First, we used KM analysis to examine the prognostic impact of these features on both GBM and LGG patients. In the TCGA, CGGA 301, CGGA 325, and GSE16011 datasets, the OS of both GBM and LGG patients was reduced in the high-risk group (Fig. 4A). Additionally, the risk scores varied significantly across different patient subgroups, with higher scores observed in patients with high-grade gliomas (Fig. 4B). In the TCGA dataset, patients with 1p/19q non-co-deletion, wild-type IDH, and unmethylated MGMT promoter had higher risk scores (Fig. 4C), which was consistent in the CGGA 301 and CGGA 325 datasets (Extended Data Fig. 1A and B). These findings demonstrated significant associations between the risk score and established clinicopathological characteristics.

### Construction and validation of the nomogram

**Fig. 5:**
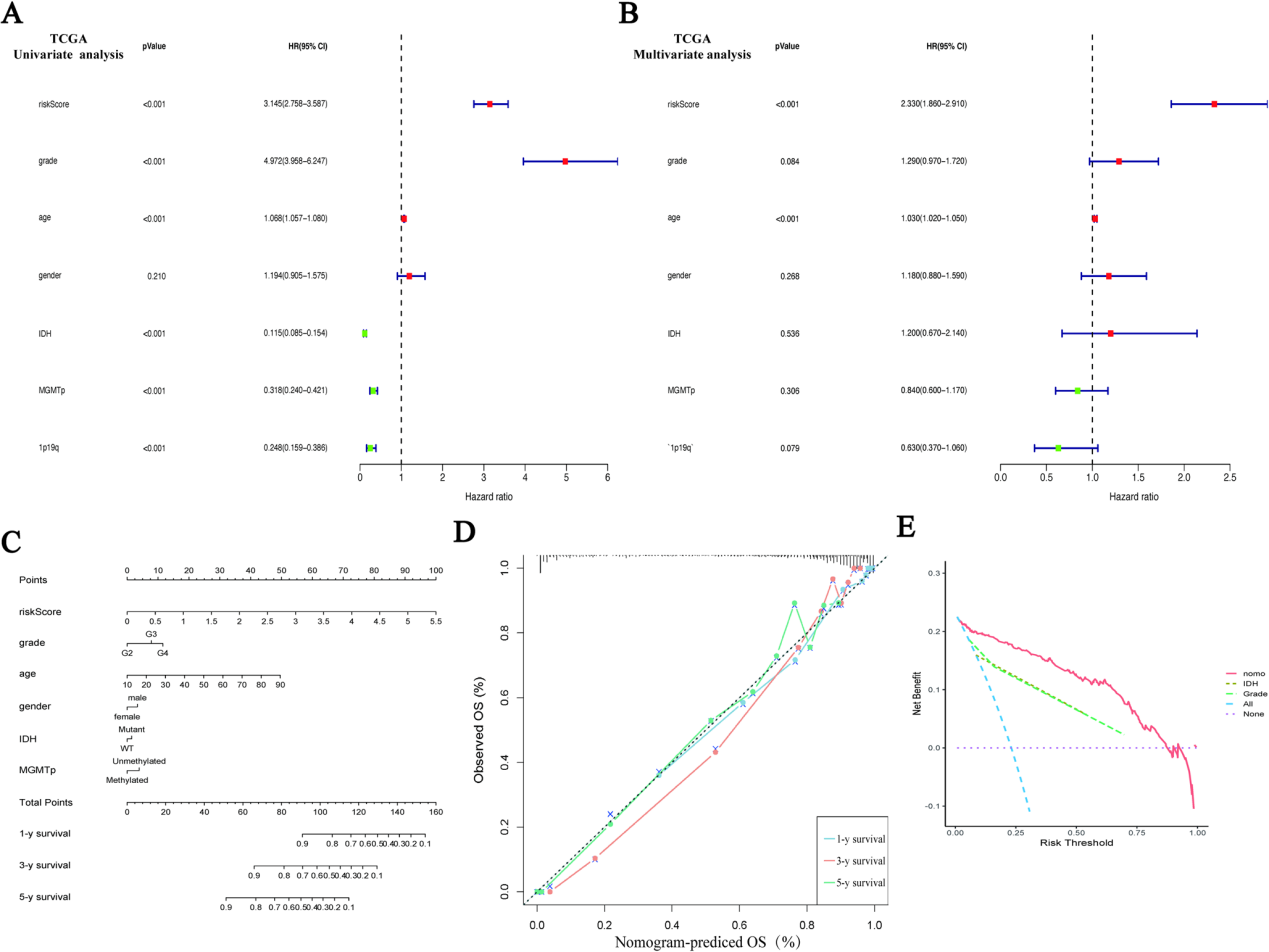
Construction and evaluation of the nomogram in the Cancer Genome Atlas (TCGA) cohort. (**A**,** B**) Forest plots of univariate (**A**) and multivariate (**B**) Cox regression analyzes in the TCGA cohort. (**C**) Nomogram constructed in the TCGA cohort based on mechanically related risk score, World Health Organization grade, age, gender, isocitrate dehydrogenase mutation status, and MGMT promoter methylation status. (**D**) Calibration plot of the nomogram based on the TCGA cohort. (E) Decision curve analysis for the overall survival nomogram in the TCGA cohort

To assess the independent prognostic value and clinical utility of the model, we performed univariate and multivariate Cox regression analyses using the R package “survival.” In the TCGA database, both univariate and multivariate analyses demonstrated that the mechanistically related risk score was significantly associated with OS [HR = 3.145, 95% confidence interval (CI: 2.758–3.587); HR = 2.330, 95% CI: 1.860–2.910]. Univariate Cox regression analysis also revealed that grade, age, IDH mutation status, MGMT promoter status, and 1p/19q co-deletion status were significantly associated with OS. Additionally, multivariate analysis indicated that age was significantly related to OS (Fig. 5A, B). These results were further validated in the CGGA 301 and CGGA 325 datasets, where univariate and multivariate analyses also confirmed that the mechanistically related risk score was a reliable independent prognostic factor for glioma OS (HR = 1.365, 95% CI: 1.268–1.469; HR = 1.120, 95% CI: 1.020–1.240; HR = 1.648, 95% CI: 1.498–1.814; HR = 1.330, 95% CI: 1.150–1.530) (Extended Data Fig. 2A and B). We developed a nomogram to predict 1-year, 3-year, and 5-year OS in the TCGA cohort based on the nine-gene risk signature, World Health Organization (WHO) grade, age, sex, IDH mutation status, and MGMT promoter methylation status, with point allocations reflecting the contribution of each feature to OS risk (Fig. 5C). The calibration curve constructed using the TCGA cohort demonstrated consistency between the predicted and actual survival times (Fig. 5D). Moreover, the DCA curve indicated that the nomogram performed well in predicting OS rates for glioma patients (Fig. 5E). Overall, these analyses supported the independent prognostic value of the nine-gene mechanosensitive risk score.

### Immune infiltration in different risk groups

**Fig. 6:**
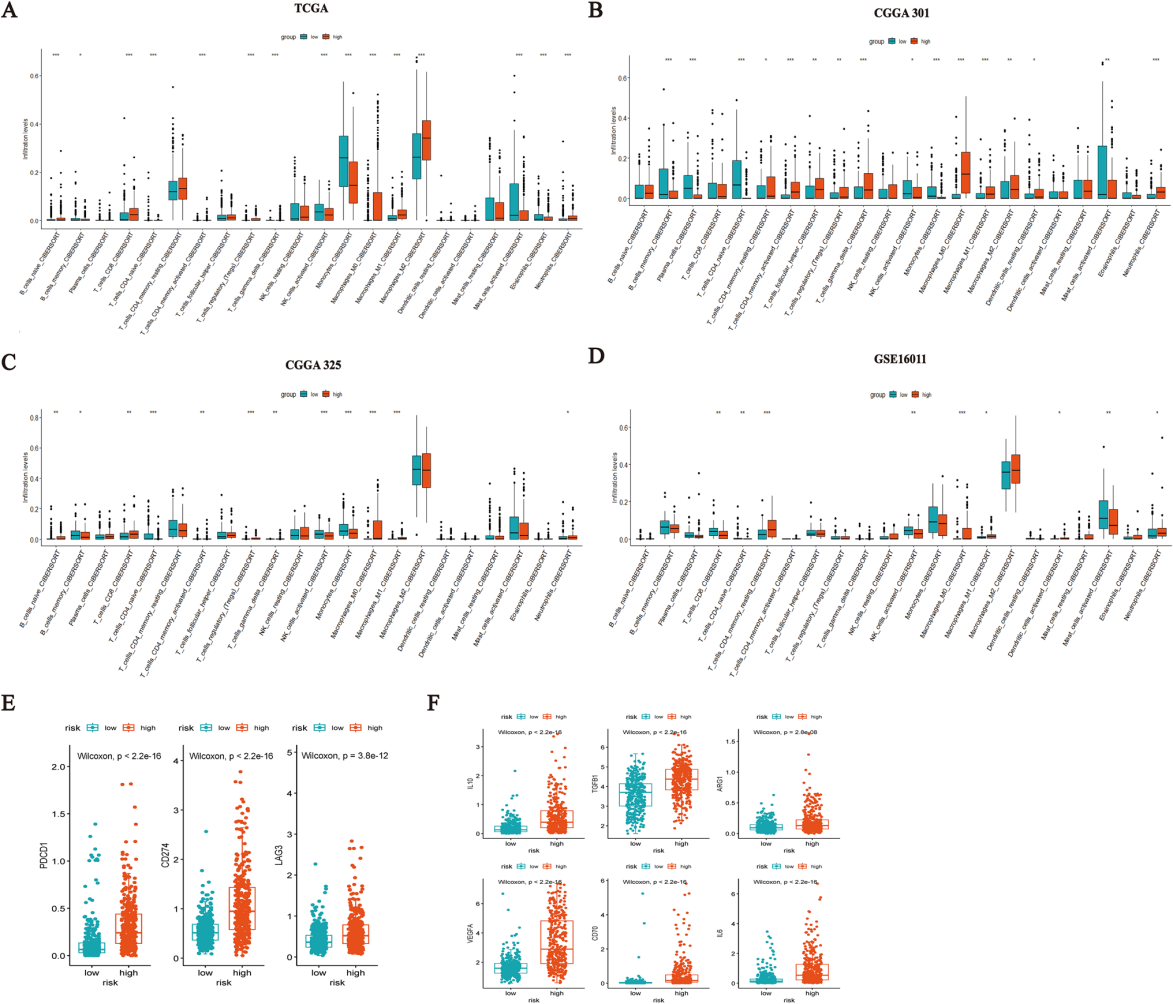
Immune cell infiltration and expression of immune factors between different risk groups. (**A**,** B**,** C**,** D**) Box plots of the infiltration levels of 22 immune cell types between different risk groups in the Cancer Genome Atlas (TCGA), Chinese Glioma Genome Atlas (CGGA) 301, CGGA 325, and GSE16011 cohorts (*P* < 0.05; **P < 0.01; ***P < 0.001). (**E**) Expression of immune checkpoints. (**F**) Expression of immunosuppressive factors

Given that KEGG analysis suggested an association between genes related to the response to mechanical stimuli and immune-related biological processes—such as the TNF signalling pathway, toll-like receptor signalling pathway, NF-kappa B signalling pathway, MAPK signalling pathway, and inflammatory mediator regulation of TRP channels—this might represent a potential prognostic mechanism underlying the glioma risk score model. It also suggested that genes related to the response to mechanical stimuli were likely to influence immune cell activity in glioma. To understand the differences in immune cell infiltration between the mechanistically defined high-risk and low-risk groups, we evaluated immune cell infiltration in each glioma sample within the TCGA cohort. Among the 22 immune cell subtypes, 9 showed significantly higher infiltration levels in the high-risk group, while 7 subtypes had higher infiltration levels in the low-risk group (Fig. 6A). Similarly, in the CGGA 301, CGGA 325, and GSE16011 cohorts, 10, 7, and 5 immune cell subtypes, respectively, exhibited higher infiltration levels in the high-risk group, whereas 6, 4, and 4 subtypes showed higher infiltration in the low-risk group (Fig. 6B, C, and D). Notably, M0 macrophages, M1 macrophages, and neutrophils exhibited higher infiltration levels in the high-risk group across all four cohorts, while naive CD4^+^ T cells and activated NK cells showed higher infiltration in the low-risk group across all four cohorts. These findings suggested that mechanistically related risk factors might promote tumor progression by influencing macrophage polarization or neutrophil activity, while mechanistically related protective factors might inhibit tumor progression through CD4^+^ T cells and NK cells. Therefore, patients in high-risk and low-risk groups might benefit from different immunotherapeutic strategies.

Understanding the immunosuppressive environment is crucial for selecting appropriate tumor immunotherapies [21]. Immune checkpoint molecules have been established as predictive factors for the efficacy of various tumor immunotherapies [22], and immunosuppressive factors in the TME can alter the fate of innate and adaptive immune cells, thereby regulating cancer cell growth and metastasis [23, 24]. We analyzed the impact of the risk score on the mRNA expression levels of three immune checkpoint molecules (*PDCD1*, *CD274*, and *LAG3*) and six immunosuppressive factors (*IL10*, *TGFβ1*, *ARG1*, *VEGFA*, *CD70*, and *IL6*) within the TCGA cohort. The results showed that all of these factors were expressed at higher levels in the high-risk group (Fig. 6E, F). Similar trends were further observed in the CGGA 325 and GSE16011 cohorts, supporting the association between the high-risk group and an immunosuppressive tumor microenvironment (Extended Data Fig. 3). In conclusion, patients in the high-risk group exhibited higher expression of immune checkpoint molecules and immunosuppressive factors, indicating a greater likelihood of immune evasion [25].

### Immunotherapy and chemotherapy responses in different risk groups

**Fig. 7:**
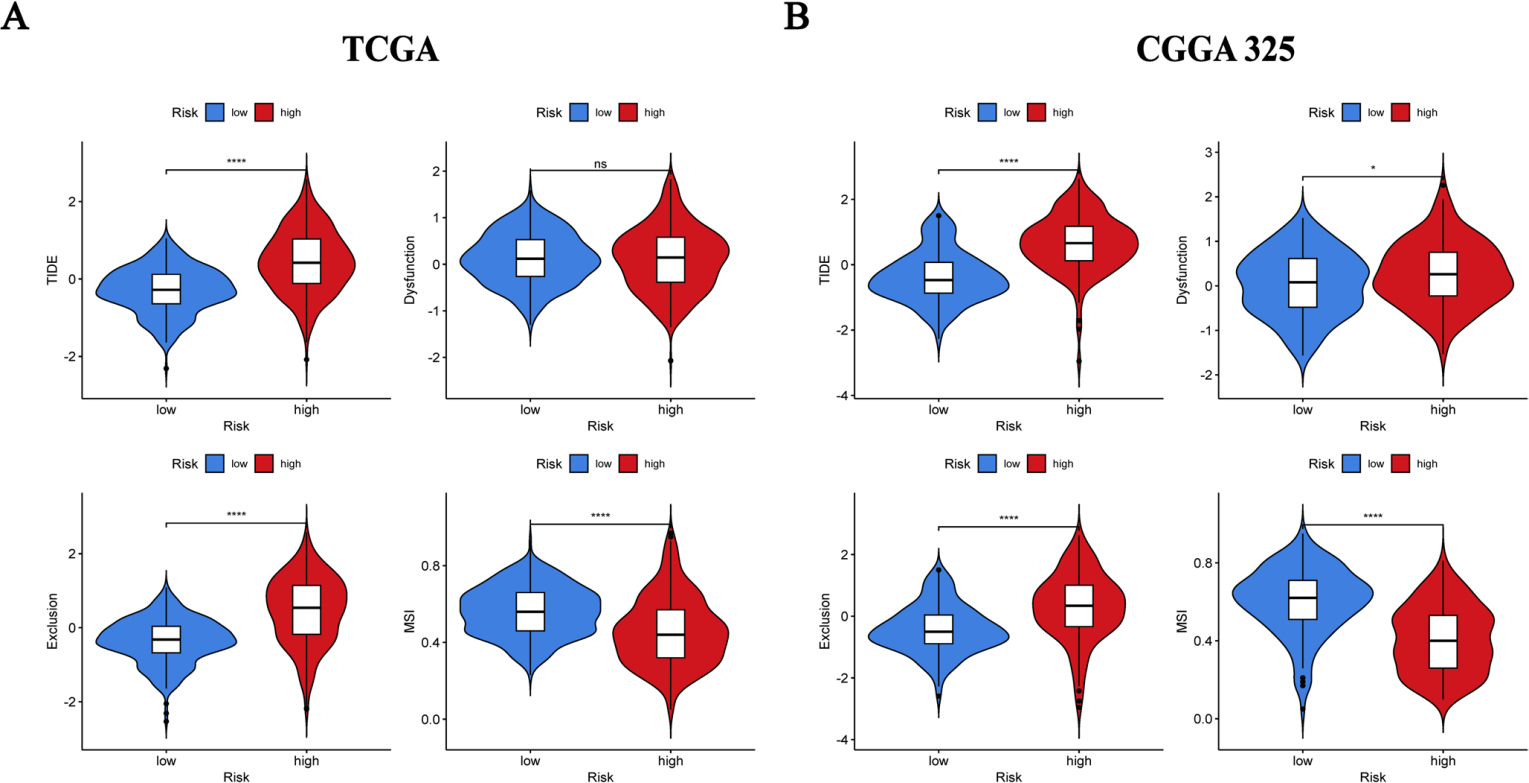
Tumor Immune Dysfunction and Exclusion (TIDE) analysis in different risk groups. (**A**) Differences in TIDE, dysfunction, exclusion, and microsatellite instability (MSI) scores between high-risk and low-risk groups in the Cancer Genome Atlas cohort. (**B**) Differences in TIDE, dysfunction, exclusion, and MSI scores between high-risk and low-risk groups in the Chinese Glioma Genome Atlas 325 cohort (*P < 0.05; ****P < 0.0001; ns: not significant)

To explore the association between the nine-gene mechanosensitive signature and predicted immune evasion, we further analyzed TIDE scores. The results showed that in the TCGA cohort, the high-risk group had significantly higher TIDE scores and immune exclusion scores, while the MSI score was lower (Fig. 7A). Similarly, in the CGGA 325 cohort, the high-risk group also showed significantly higher TIDE, immune dysfunction, and immune exclusion scores, with a lower MSI score (Fig. 7B). These findings suggested that patients in the high-risk group had an increased predicted potential for immune evasion, which may be associated with reduced responsiveness to ICIs.

**Fig. 8:**
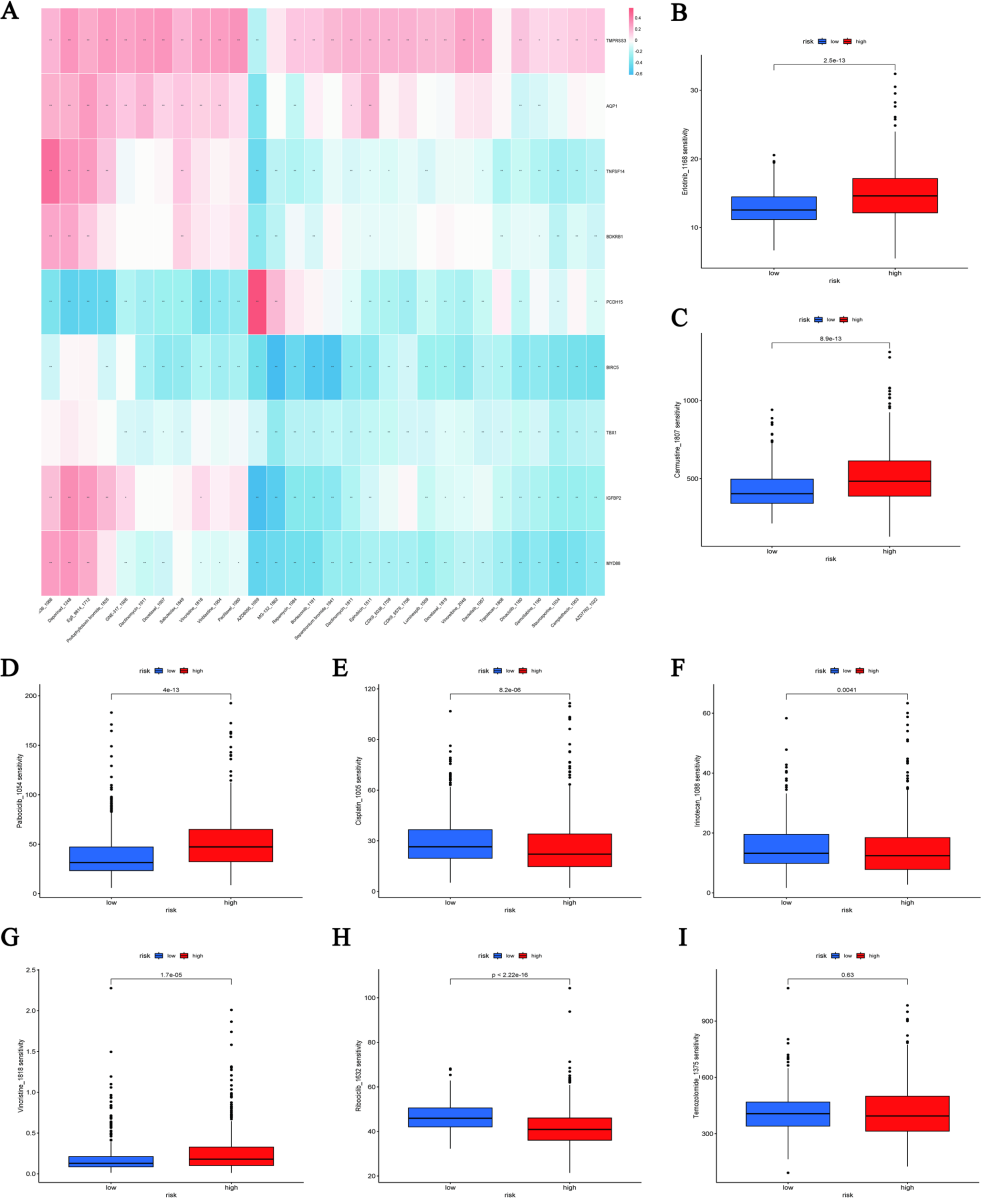
Chemotherapy drug sensitivity analysis in different risk groups. (**A**) Correlation analysis between the nine genes related to the response to mechanical stimuli and commonly used chemotherapeutic drugs for glioma (*P < 0.05; **P < 0.01). Box plots of IC50 values between high- and low-risk groups in the Cancer Genome Atlas dataset for Erlotinib (**B**), Carmustine (**C**), Palbociclib (**D**), Cisplatin (**E**), Irinotecan (**F**), Vincristine (**G**), Ribociclib (**H**), and Temozolomide (**I**). Lower IC50 indicates higher drug sensitivity (Wilcoxon test)

Glioblastoma exhibits varying sensitivity to different chemotherapy drugs, with response rates ranging from 40% to 80%. To investigate the impact of the mechanistically related nine-gene signature on glioma chemosensitivity, we analyzed the correlation between these genes and commonly used chemotherapeutic agents for glioma. The results revealed differential correlations between these genes and various drugs (Fig. 8A). Additionally, we used IC50 values to measure differences in chemosensitivity between the high-risk and low-risk groups. It was found that the high-risk group had higher IC50 values for erlotinib (Fig. 8B), carmustine (Fig. 8C), palbociclib (Fig. 8D), and vincristine (Fig. 8G), indicating that the low-risk group was more sensitive to these drugs. Conversely, the high-risk group had lower IC50 values for cisplatin (Fig. 8E), irinotecan (Fig. 8F), ribociclib (Fig. 8H), and temozolomide (Fig. 8I), suggesting greater sensitivity to these agents in the high-risk group. Notably, cisplatin could stimulate T-lymphocyte function to enhance immune responses, providing some level of immunostimulatory effect [26], while temozolomide was known to enhance T-lymphocyte proliferation and function, which was often combined with immunotherapy for glioma treatment [27]. These findings suggested that the nine-gene risk signature was associated with predicted drug sensitivity patterns, which require further experimental and clinical validation.

### Cell type identification and intercellular communication in glioma tissues

**Fig. 9:**
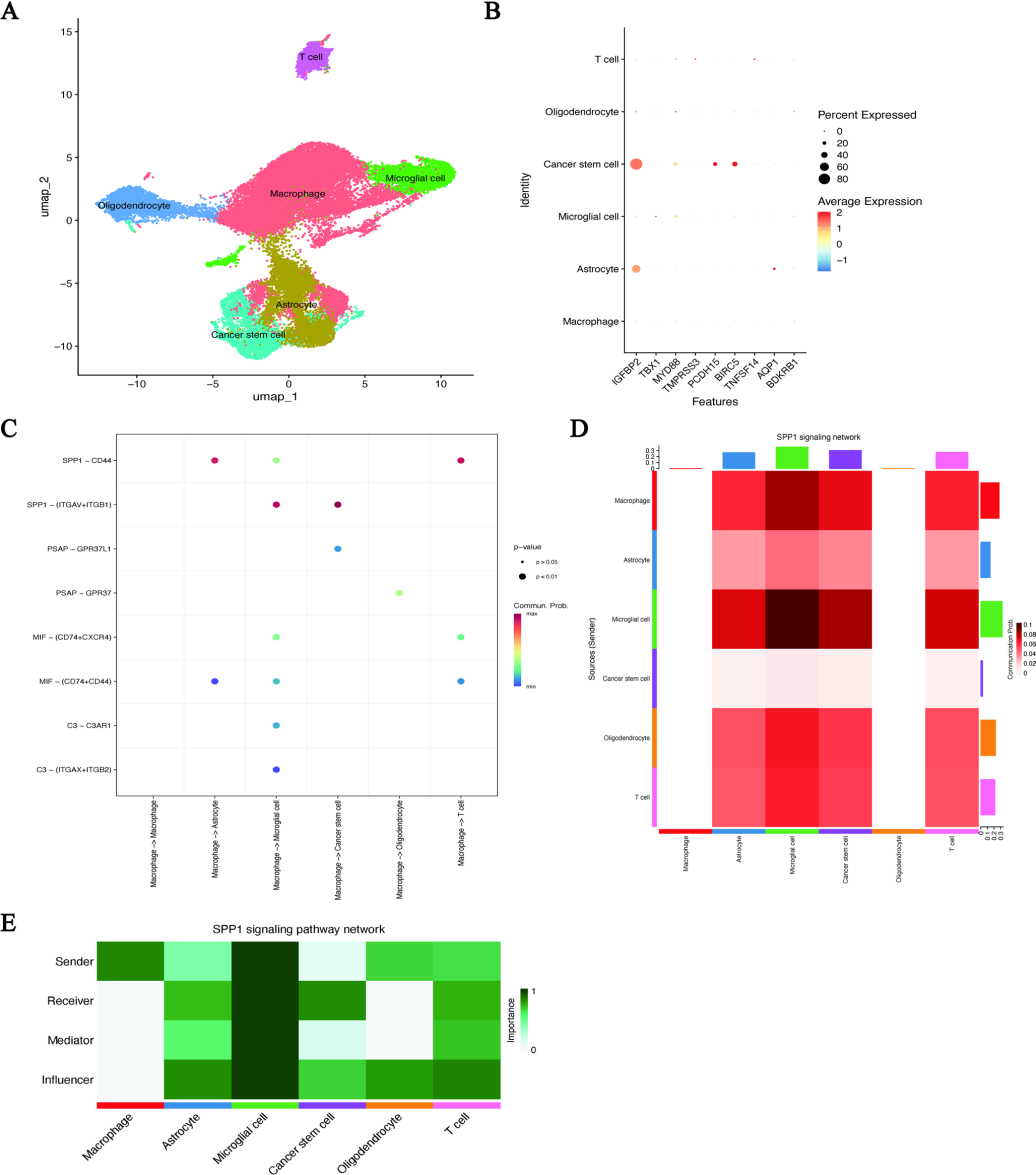
Single-cell transcriptome analysis of the nine mechanical stimuli-related gene signature and cell–cell communication between different cell types. (**A**) Clustering and annotation of single-cell data. (**B**) Expression of the nine genes related to the response to mechanical stimuli across six cell types in glioma. (**C**) Bubble plot showing all significant ligand-receptor pairs. (**D**) Heatmap displaying the secreted phosphoprotein 1 (SPP1) signalling pathway among different cell types in glioma. (**E**) Heatmap illustrating the specific roles of different cell types in the SPP1 signalling pathway

After mitochondrial gene quality control, we included a total of 37,090 cells in our analysis. Using the Seurat package, we performed cell clustering and classified glioma cells into six categories based on cell markers from the Cell Marker database (Supplementary Table 9): astrocytes, microglial cells, cancer stem cells, oligodendrocytes, T cells, and macrophages (Fig. 9A). We further explored the expression of the nine genes responsive to mechanical stimuli across these six cell types. IGFBP2 was primarily expressed in astrocytes and cancer stem cells; PCDH15 and BIRC5 were also mainly expressed in cancer stem cells, while AQP1 was predominantly expressed in astrocytes. TBX1, MYD88, TMPRSS3, TNFSF14, and BDKRB1 showed relatively low expression across all six cell types (Fig. 9B). We then investigated the interactions between these cell types.

Among these interactions, secreted phosphoprotein 1 (SPP1) - CD44 and SPP1-(ITGAV + ITGB1) were identified as potentially playing key roles in cell communication among macrophages, T cells, microglial cells, and cancer stem cells in glioma (Fig. 9C). The heatmap further illustrated the communication probability in the SPP1 signalling pathway among different cell groups and highlighted the roles each cell type played in this pathway (Fig. 9D, E). These findings suggested that the SPP1 signalling pathway might be a crucial mechanism through which mechanistic signals regulate the complex immune microenvironment.

### SPP1 expression correlates with the mechanosensitive risk score

Given that SPP1‑related signaling was identified as a potential immune‑regulatory pathway in our single‑cell analysis, we next examined whether SPP1 expression correlated with the nine‑gene risk score in bulk transcriptomic cohorts. Spearman correlation analysis revealed a significant positive correlation between the risk score and SPP1 expression in the TCGA (*R* = 0.680, *p* < 0.001), CGGA‑301 (*R* = 0.617, *p* < 0.001), CGGA‑325 (*R* = 0.665, *p* < 0.001), and GSE16011 (*R* = 0.620, *p* < 0.001) cohorts (Extended Data Fig. 4). These findings establish a direct link between the mechanosensitive risk signature and SPP1 expression, supporting the potential involvement of SPP1‑mediated pathways in the immune microenvironment differences observed between risk groups.

### Validation of RNA and protein expression levels of the nine genes responsive to mechanical stimuli in glioma

**Fig. 10:**
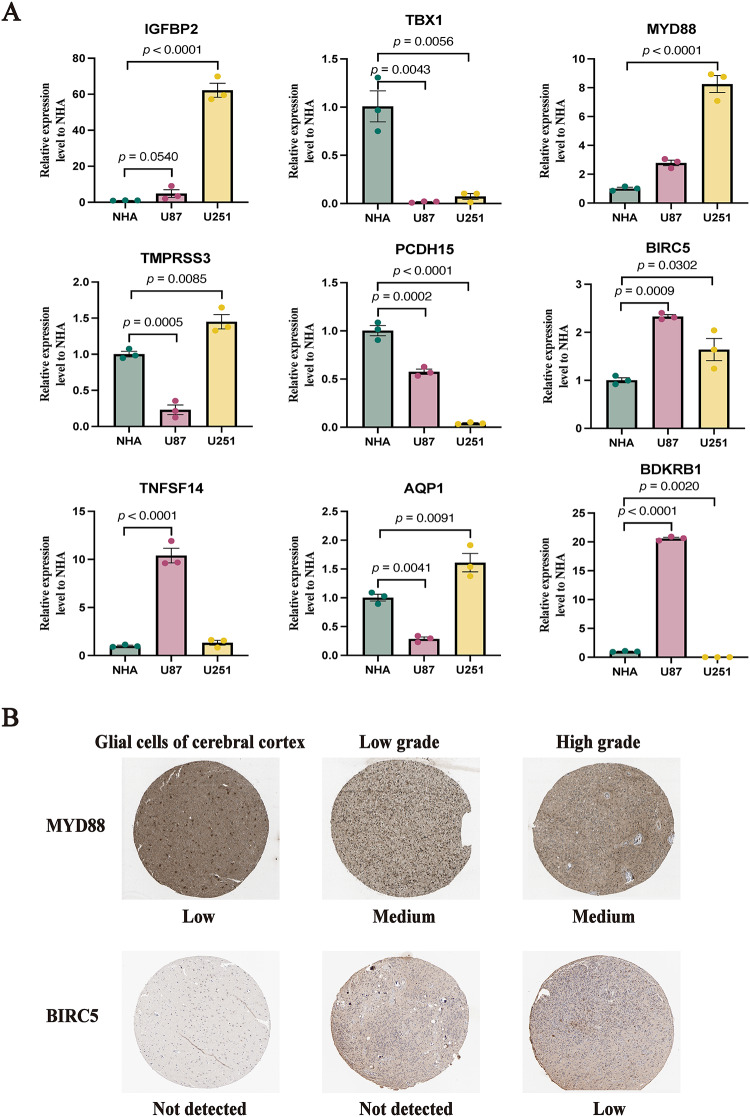
Expression levels of the nine genes related to the response to mechanical stimuli in glioma. (**A**) Quantitative reverse transcription PCR analysis comparing the expression levels of *IGFBP2*, *TBX1*, *MYD88*, *TMPRSS3*, *PCDH15*, *BIRC5*, *TNFSF14*, *AQP1*, and *BDKRB1* in NHA, U87, and U251 (mean, *n* = 3). (**B**) The expression profiles of the proteins encoded by *MYD88* and *BIRC5* in glial cells of cerebral cortex, low-grade and high-grade glioma tissues using clinical specimens from the Human Protein Profiles (expression levels determined by antibody staining)

We assessed the RNA expression levels of the nine genes responsive to mechanical stimuli in the human astrocyte cell line NHA and the glioma cell lines U87 and U251. The results revealed that the glioma protective factors *TBX1* and *PCDH15* were indeed expressed at lower levels in U87 and U251 compared to NHA, whereas the glioma risk factors *IGFBP2*, *MYD88*, *TMPRSS3*, *BIRC5*, *TNFSF14*, *AQP1*, and *BDKRB1* showed higher expression levels in U87 and/or U251 than in NHA (Fig. 10A). These findings suggested that our analytical approach could serve as a potential model for identifying protective or risk factors in tumors, and the nine genes responsive to mechanical stimuli had potential application value.

To further validate their expression in glioma tissues, we used the Human Protein Atlas (HPA) database (https://www.proteinatlas.org/) to examine the protein expression levels of IGFBP2, TBX1, MYD88, TMPRSS3, PCDH15, BIRC5, TNFSF14, AQP1, and BDKRB1 in glioma. Among these, the risk factors IGFBP2 and TMPRSS3 and the protective factors TBX1 and PCDH15 were not detected. For the remaining risk factors, MYD88 was found to be expressed at higher levels in both high-grade gliomas and LGGs compared to normal cerebral cortex tissue, and BIRC5 was expressed at higher levels in high-grade gliomas compared to LGGs and normal cerebral cortex tissue (Fig. 10B). No significant differences were observed in the expression levels of TNFSF14, AQP1, and BDKRB1 across the different tissues (Extended Data Fig. 5). Therefore, *MYD88* and *BIRC5* might serve as novel targets for future glioma therapies.

## Discussion

The role of mechanical stress in various tumors, such as the stiffness and sensing capabilities of the ECM, has gained increasing attention [28]. These mechanical properties have been shown to induce and influence biological behaviours, particularly in cancers such as breast cancer [29, 30], prostate cancer [31, 32], colorectal cancer [33], and lung cancer [34]. Gliomas reside in brain tissue with relatively low mechanical resistance, which facilitates their invasion and other poor prognostic characteristics [35]. Currently, 11 external mechanical forces and 2 TME forces have been identified as being associated with GBM migration, invasion, and treatment resistance [36]. For instance, tensile forces applied to GBM could promote the secretion of tenascin-C, increasing ECM stiffness and, in turn, enhancing tumor invasion [16]. Additionally, mechanical compression forces on GBM could induce epigenetic signalling, further increasing tumor aggressiveness [37]. Environmental pressure could also enhance GBM invasiveness by upregulating caveolin-1 or epithelial-mesenchymal transition (EMT) signalling pathways [38, 39]. However, the impact of brain tissue stiffness on glioma is complex and remains poorly understood, with a lack of relevant preclinical studies. Therefore, analysing mechanosensitive genes may be a key approach to identifying the mechanical signals that most significantly impact glioma prognosis. In this study, we constructed a glioma risk signature using genes related to the response to mechanical stimuli and identified individuals at high and low risk for poor prognostic outcomes. This approach may provide complementary information for molecular risk stratification and clinical outcome prediction.

We identified 399 genes related to the response to mechanical stimuli from five mechanosensitive gene sets. In the TCGA dataset, we performed a Cox proportional hazards model and KM survival analysis to identify 292 survival-related genes. GO and KEGG analyses confirmed that these genes were involved in responses to mechanical stimuli, mechanoreceptor organ development, and several immune-related signalling pathways, such as the TNF signalling pathway [40], toll-like receptor signalling pathway [41], NF-kappa B signalling pathway, MAPK signalling pathway [25], inflammatory mediator regulation of TRP channels [42, 43], etc. Further LASSO regression and multivariate Cox regression analyses using the AIC method identified nine key genes related to the response to mechanical stimuli (*IGFBP2*, *TBX1*, *MYD88*, *TMPRSS3*, *PCDH15*, *BIRC5*, *TNFSF14*, *AQP1*, and *BDKRB1*). Prognostic feature analysis revealed that *TBX1* and *PCDH15* were protective factors for glioma survival, whereas *TMPRSS3*, *BIRC5*, *TNFSF14*, *AQP1*, and *BDKRB1* were risk factors for glioma survival.

The mechanosensitive signature demonstrated consistent prognostic value across three independent validation cohorts and remained independently associated with overall survival after adjustment for established clinicopathological factors. The nomogram further suggested that integrating the risk score with conventional clinical variables may improve individualized prognostic assessment.

Experimental evidence showed that the nine genes related to the response to mechanical stimuli played different roles in glioma. For instance, TMPRSS3 had been shown to promote glioma proliferation, migration/invasion, and inhibit apoptosis [44]. Conversely, PCDH15 could suppress glioma progression in vivo by inhibiting Wnt signalling, consistent with our analysis identifying it as a protective factor in glioma [45]. Additionally, IGFBP2, MYD88, BIRC5, TNFSF14, and AQP1 were involved in regulating the complex glioma immune microenvironment. For example, IGFBP2, a cancer-associated fibroblast (CAF)-related gene, was considered a glioma tumor promoter, enhancing microglial migration [46] and promoting glioma progression by inducing M2 macrophage polarization [47]. MYD88 expression was associated with a malignant phenotype and poor prognosis, regulating innate immunity through direct effects on Toll-like receptors, cytokine receptors, NF-kappa B signalling, and MAPK signalling pathways [48]. Patients with higher MYD88 expression also showed greater M2 macrophage infiltration [49]. BIRC5 was associated with glioma migration and immune infiltration [50], and it promoted glioma stemness and the EMT process [51]. TNFSF14, a novel immune checkpoint molecule, played a role in establishing the immune microenvironment [52]. Targeting TNFSF14 could alter vascular phenotypes, enhance effective antitumor T-cell responses, and prolong survival in glioma patients [53]. AQP1 contributed to glioma migration, invasion, and angiogenesis [54, 55]. These observations suggest that the genes included in the signature may be biologically relevant to glioma progression and immune regulation, although their mechanistic roles require further validation.

Glioma, as an aggressive brain tumor, exhibits strong resistance to immunotherapy [53]. In the complex TME, mechanical cues not only influence the behavior of cancer cells but also significantly impact the tumor immune response [56]. Understanding the interactions between mechanical forces and immune cell behavior can shed light on cancer progression, deepen our understanding of TME mechanobiology, and is crucial for developing effective therapies for solid tumors [57]. A recent review has highlighted that glioma-associated epilepsy is shaped by molecular subtype, glutamatergic dysregulation, neuroinflammation, and interactions between tumor and non-neoplastic cells, further underscoring the close links among glioma biology, neural activity, and the tumor microenvironment [58]. Our KEGG analysis of genes related to the response to mechanical stimuli indicated their involvement in immune signalling pathways. Consequently, we further analyzed immune cell infiltration levels across different risk groups. We found that M0 macrophages, M1 macrophages, and neutrophils exhibited higher infiltration levels in the high-risk group, whereas naive CD4^+^ T cells and activated NK cells showed higher infiltration levels in the low-risk group across all four cohorts. These findings suggest that genes related to the response to mechanical stimuli may be associated with glioma immune microenvironment heterogeneity and may provide exploratory clues for future immunological and therapeutic studies.

To further assess the immunotherapeutic efficacy in glioma patients across different risk groups, we performed TIDE analysis. The results indicated that the high-risk group had an increased potential for immune evasion, suggesting that these patients might respond poorly to ICIs. Conversely, the low-risk group might respond better to immunotherapy. Additionally, we observed differences in drug sensitivity between the high- and low-risk groups. The low-risk group was more sensitive to Erlotinib, Carmustine, Palbociclib, and Vincristine, whereas the high-risk group was more sensitive to Cisplatin, Irinotecan, Ribociclib, and Temozolomide. Together, these findings suggest that the nine-gene risk signature may provide exploratory information related to predicted immunotherapy response and drug sensitivity, but these predictions require experimental and clinical validation before they can guide treatment decisions.

Single-cell analysis suggested that the SPP1 signalling pathway might represent a potential communication pathway involved in regulating the complex glioma immune microenvironment. In addition, the risk score was consistently positively correlated with SPP1 expression across four independent bulk transcriptomic cohorts, providing further support for an association between the mechanosensitive signature and SPP1-related immune communication. SPP1 appeared to mediate cell communication within the TME among macrophages, T cells, microglial cells, and cancer stem cells through its ligands CD44 and ITGAV/ITGB1. SPP1, also known as osteopontin, was a mechanosensitive gene involved in mechanotransduction pathways [59, 60] and served as a crucial regulator of the complex interactions between cancer cells and the TME [61]. Its positive association with poor prognosis in glioma [62] further supporting a potential association between mechanosensitive signaling and immune microenvironment remodeling in glioma.

Existing studies suggested that SPP1-CD44 and SPP1-ITGAV/ITGB1 interactions were associated with immune suppression within the GBM microenvironment. The interaction between SPP1 and CD44 on macrophages could promote the polarization of macrophages from the M1 to the M2 phenotype [63], while the activation of naive T cells into CD8^+^ cytotoxic T cells might be inhibited by SPP1 ligands [64], leading to immune suppression. Additionally, experiments had shown that SPP1 targeted the ITGAV/ITGB1 receptors on macrophages, influencing M2 polarization and subsequently activating the downstream AKT signalling pathway, which was associated with poor prognosis in GBM patients [65]. Our analysis, along with existing research, suggested that SPP1, in conjunction with CD44 and ITGAV/ITGB1, may represent potential molecules involved in tumor–immune communication, warranting further functional validation. However, the mechanisms by which these molecules affect other cells within the TME warrant further investigation.

We further validated the expression of the nine genes related to the response to mechanical stimuli in U87 and U251 glioma cell lines. Compared with those to NHA, their expression levels were generally consistent with our predictions. However, in the HPA, only MYD88 and BIRC5 were confirmed to align with our predictions. These findings provide preliminary experimental support for part of the analytical model, although its findings were primarily based on public databases. Therefore, additional in vivo and in vitro studies are needed to confirm our results. The roles and mechanisms of these nine genes related to the response to mechanical stimuli in glioma initiation and progression warrant further investigation.

Although the mechanical properties of gliomas remain an area of ongoing investigation [12], the present study integrates bulk transcriptomic analysis, external validation, and single‑cell transcriptomic analysis to characterize mechanosensitive gene expression patterns associated with glioma prognosis and the immune landscape. Nevertheless, several limitations should be acknowledged. First, most analyses were based on retrospective public transcriptomic datasets, and differences in sequencing platforms, clinical annotation, treatment background, follow‑up duration, and molecular classification may introduce unavoidable heterogeneity. Second, although the mechanosensitive gene signature was associated with survival and immune features across multiple cohorts, these associations are correlative and should not be interpreted as evidence of causality. Third, computational estimates of immune infiltration, TIDE scores, pathway activity, and drug sensitivity provide indirect predictions and require validation using orthogonal approaches such as immunohistochemistry, spatial transcriptomics, single‑cell profiling, functional assays, or prospective clinical datasets. Fourth, the incremental prognostic value of this signature over established markers, including WHO grade, IDH status, 1p/19q codeletion, MGMT promoter methylation, age, treatment, and performance status, requires further evaluation in clinically standardized cohorts. Finally, additional in vivo and in vitro studies are needed to determine whether the selected mechanosensitive genes actively regulate glioma progression, immune remodeling, or therapy response. In this context, ongoing prospective biomarker studies in glioblastoma, such as those investigating olfactory function as a prognostic factor, also exemplify the value of longitudinal and multidimensional approaches for identifying clinically useful prognostic markers [66]. In conclusion, this study identified a mechanosensitive gene signature that is associated with survival and immune landscape heterogeneity in glioma and may provide complementary information for risk stratification.

## Supplementary Information

Below is the link to the electronic supplementary material.

Supplementary Material 1

Supplementary Material 2

## Data Availability

The transcriptomic datasets analyzed in this study are publicly available from The Cancer Genome Atlas (TCGA) through the UCSC Xena platform, the Chinese Glioma Genome Atlas (CGGA), and the Gene Expression Omnibus (GEO) database under accession numbers GSE16011 and GSE271959. All datasets were accessed in accordance with the respective data usage policies.

## Abbreviations

TME: Tumor microenvironment
ECM: Extracellular matrix
TCGA: The Cancer Genome Atlas
GEO: Gene Expression Omnibus
CGGA: Chinese Glioma Genome Atlas
GO: Gene Ontology
KEGG: Kyoto Encyclopedia of Genes and Genomes
KM: Kaplan-Meier
LASSO: Least absolute shrinkage and selection operator
OS: Overall survival
ROC: Receiver operating characteristic
IDH: Isocitrate dehydrogenase
DCA: Decision curve analysis
ICIs: Immune Checkpoint Inhibitors
TIDE: Tumor Immune Dysfunction and Exclusion
GDSC-V2: Genomics of Drug Sensitivity in Cancer V2
qRT-PCR: Quantitative reverse transcription PCR
AUC: Area under the curve
HRs: Hazard ratios
GBM: Glioblastoma
LGG: Low-grade glioma
CI: Confidence interval
WHO: World Health Organization
MSI: Microsatellite instability
SPP1: Secreted phosphoprotein 1
HPA: Human Protein Atlas
EMT: Epithelial-mesenchymal transition
