# Supplementary material for: A mechanosensitive gene signature associated with immune landscape and survival in glioma: a multi-cohort analysis
Source: Neurol Res Pract. 2026 Aug 7;8(1):62. doi: 10.1186/s42466-026-00515-2 (PMC13452127; doi:10.1186/s42466-026-00515-2)
Supplement: Supplementary file 1 [file 42466_2026_515_MOESM2_ESM.docx]

**Supplemental Information**

**Genetic Signatures Responsive to Mechanical Stimuli Reveal the Guiding Role of Mechanical Signals in The Prognosis and Treatment of Glioma Patients**

**Extended Data Fig. 1: Mechanical feature risk scores across different clinicopathological characteristics in the Chinese Glioma Genome Atlas (CGGA) 325 and CGGA 301 cohorts**

(A) Risk scores stratified by 1p19q co-deletion status, isocitrate dehydrogenase (IDH) mutation status, and MGMT promoter methylation status in the CGGA 325 cohort. (B) Risk scores stratified by IDH mutation status and MGMT promoter methylation status in the CGGA 301 cohort (Wilcoxon test).

**Extended Data Fig. 2: Univariate and multivariate independent prognostic analyses in the Chinese Glioma Genome Atlas (CGGA) 301 and CGGA 325 cohorts**

(A, B) Forest plots of univariate (A) and multivariate (B) Cox regression analyses in the CGGA 301 cohort. (C, D) Forest plots of univariate (C) and multivariate (D) Cox regression analyses in the CGGA 325 cohort.

**Extended Data Fig. 3: Analysis of immune checkpoints and immunosuppressive factors in the Chinese Glioma Genome Atlas (CGGA) 325 and GSE16011 cohorts**

(A, B) mRNA expression levels of *PDCD1*, *CD274*, and *LAG3* in high-risk and low-risk groups in the CGGA 325 and GSE16011 cohorts. (C, D) mRNA expression levels of *IL10*, *TGFβ1*, *ARG1*, *VEGFA*, *CD70*, and *IL6* in high-risk and low-risk groups in the CGGA 325 and GSE16011 cohorts.

**Extended Data Fig. 4: Correlation between the nine-gene risk score and SPP1 expression in bulk glioma cohorts.**

Spearman correlation analyses between the risk score and SPP1 expression in the TCGA, CGGA-301, CGGA-325, and GSE16011 cohorts. Spearman correlation coefficients (R) and corresponding *P* values are shown in panels.

**Extended Data Fig. 5: The expression profiles of the proteins encoded by TNFSF14, AQP1, and BDKRB1 in glial cells of cerebral cortex, low-grade, and high-grade glioma tissues using clinical specimens from the Human Protein Profiles (expression levels determined by antibody staining)**
